# Computational Biology in Argentina

**DOI:** 10.1371/journal.pcbi.0030257

**Published:** 2007-12-28

**Authors:** Sebastian Bassi, Virginia González, Gustavo Parisi

**Affiliations:** University of California San Diego, United States of America

Computational biology is an interdisciplinary science bred from fields as disparate as mathematics, chemistry, statistics, physics, biology, and computer science. Although the exact definition of computational biology is far from being precise and unambiguous, it is a fact that hundreds of scientists around the world have been increasingly using skills from the above-mentioned fields to approach different biological questions. It is not our aim to elucidate here the definition of computational biology and its differences from related fields such as bioinformatics. However, to review the state of this discipline in Argentina, we need at least a working definition. In this sense, any interdisciplinary research in which the main interest is in studying biological problems and where the working hypothesis can be tested by means of simulation and computational modeling will be considered as belonging to the computational biology field or at least as employing a computational biology approach.

It is interesting to note that computational biology research can be implemented in two different ways depending on how the “interdisciplinary” nature of this field is assembled. On one hand, a scientist with formal training in a given field (for example, a molecular biologist) could acquire other skills (such as programming or mathematical training) in his attempt to answer a given biological question. Alternatively, working teams made up of members specializing in different fields may work together to reach a scientific explanation of a problem. We think that the first approach is more common among the scientific community because it depends entirely on the scientist's desire to discover an explanation for their problems and on their capacity to explore different areas of science. The second approach is generally dependent on the availability of research and development programs, funded by public or private resources, that favor collaborations between different research institutions to form multidisciplinary teams. We will see that the first approach is more common in Argentina.

This paper summarizes the state of the art of computational biology in Argentina. Our aim is to offer as broad a view as possible of the different groups of scientists and their main research interests. Also, we present a brief review of educational, research, and development policies related to the field. We hope that this review may encourage overseas researchers to contact and collaborate with Argentinean teams, as well as organizing and facilitating the exchange of information between researchers in our country. However, this review will probably be far from complete, due to the lack of centralized information on computational biology, and for this reason we apologize for any potential omissions. (For author information, see Box 1.)

**Box 1. Authors' Biographies**

**Gustavo Parisi, Ph.D.**, is the Structural Bioinformatics Group leader and professor at the Universidad Nacional de Quilmes, Buenos Aires, Argentina. He is also a researcher at the Consejo Nacional de Investigaciones Científicas y Técnicas (CONICET). For his Ph.D. thesis, he developed a model of protein molecular evolution which takes into account protein structure to simulate sequence divergence. His principal research interest is focused on the introduction of evolutionary information in the development of bioinformatics tools to study protein structure.

**Virginia González**, a graduate student in biotechnology, is part of the Structural Bioinformatics Group at the Universidad Nacional de Quilmes, Buenos Aires, Argentina. She is a member of the DNALinux developer team and is currently working on her doctorate in Sequential, Structural, and Dynamic Characterization of Allergenic Proteins. She is also part of the Latin America Solanaceae Genome Project (LAT-SOL) working on the sequence annotation of the tomato mitochondrial genome.

**Sebastian Bassi** worked for five years as a genome database curator at Advanta Seeds, a plant biotechnology company. He was in charge of the molecular marker database for all crops in the enterprise. He is the leader of the DNALinux bioinformatics distribution and has developed several Web-based utilities for bioinformatics analysis. He is part of the developer team for Biopython (http://www.biopython.org/), and is currently writing a book on Python for Bioinformatics and doing bioinformatics support for the tomato mitochondrial genome sequencing project, which is part of the Latin America Solanaceae Genome Project (LAT-SOL).

## Computational Biology Research in Argentina

Based on the academic background of principal team members, we can differentiate two main types of computational biology groups in Argentina. On one hand, there are research groups coming from areas such as chemistry and physics. In general, these groups have a solid background in mathematics and statistics and they can easily adapt or develop computational techniques. Several of these groups have directed their interest toward biological problems in the last few years. Such groups generally offer atomic or molecular explanations using molecular modeling techniques based in quantum, classical, or hybrid quantum/classical mechanics. Also, and expanding beyond the molecular level, we found groups engaged in modeling metabolisms, signaling pathways, and simulating learning processes or auditory physiology. Some teams work in close collaboration with experimental groups to test their predictions.

Among these types of groups, we find the group headed by Adriana Pierini at the Departamento de Química Organica, Universidad Nacional de Córdoba. This group has expertise in the application of molecular modeling techniques, based on electron-structure methods, to the study of different mechanisms of relevance in physical–organic chemistry. These studies have been mainly focused on modeling reactions that occur through single electron transfer and are currently used to model oxidation reactions in peroxidases [1]. At Universidad Nacional de Quilmes, Juliana Palma is working on methods to study proton transfer reactions in solution and enzymes. This group uses electron-structure calculations, quantum mechanics, and molecular mechanics calculations, standard molecular dynamics, and mixed quantum/classical molecular dynamics. Currently, they are studying the proton transfer process that occurs during the rate-limiting step in the oxidation of methylamine in the enzyme methylamine dehydrogenase [2].

Dario Estrin's group focuses on the study of different processes in hemoproteins using computational classical molecular dynamics and hybrid quantum/classical techniques [3,4]. The main focus in the group of Ricardo Enriz, at Universidad Nacional de San Luis, is the study of structural-activity relationships using molecular modeling. Recently, they used molecular dynamics to study guanacona, a plant metabolite with anti-tumoral properties [5], along with conformational analysis of peptides with antifungal activity [6]. Molecular dynamics has also been used to simulate protein denaturation by pressure [7] in the Instituto de Física de Líquidos y Sistemas Biológicos headed by Raul Grigera. The group led by Julián Echave is interested in the physical and biological factors that determine protein properties. They apply a variety of methods, from performing classical and quantum mechanical simulations to developing models of the evolutionary divergence of protein sequence, structure, and dynamics [8–10] at the Instituto de Investigaciones Fisicoquímicas Teóricas y Aplicadas, Universidad Nacional de La Plata-CONICET. At the Universidad Nacional de Quilmes, the group headed by Sebastián Fernández Alberti focuses their research in protein and dendritic (light harvesters) dynamics using classical and hybrid quantum/classical simulations. They are interested in processes of energy transfer, vibrational relaxation, and intramolecular energy redistribution. In addition, they study the effects that mutations have on protein dynamics and function, including cold adaptation, ligand affinities, allosterism, and conformational diversity [11,12].

There are several groups in the Departamento de Fisica, Facultad de Ciencias Exactas, Universidad Nacional de Buenos Aires who are using computational tools to understand biological problems. More specifically, one of these groups, headed by Silvina Ponce Dawson, works on the mathematical modeling of intracellular calcium signals and on the development of novel numerical techniques to infer quantitative information from microscopy images [13,14]. Another group works on birdsong as a model system to study the process of learning. This group does both experimental and theoretical work in which computers are used to simulate the observed dynamics [15,16]. Mariano Sigman's Integrative Neurodynamics Group also uses computational tools both for modeling and to analyze experimental data. They are also following a systems biology approach to infer the properties of some signaling mechanisms in yeast, and to work on genomics developing new software tools to extract information from large databases. Also, the Universidad Nacional de Quilmes houses the Laboratorio de Acústica y Percepción Sonora, headed by Manuel Eguía. This group is concerned with simulation of auditory periphery models in mammals, calcium dynamics in ribbon synapses of inner hair cells, and small networks of anteroventral cochlear nucleus cells. They also study the envelope encoding of natural stimuli in the cochlear nucleus and inferior colliculus.

The other type of computational biology research groups in Argentina consists of teams derived from biological disciplines such as biology, biochemistry, and agriculture. Many of these groups are involved in diverse genome sequencing projects and in the corresponding databases' curation. Most of the organisms involved in these studies are relevant to local health or agricultural issues. Other groups are involved in the development of bioinformatics tools for the characterization of sequence and protein structure or for the analysis of microarrays, while others are working in evolutionary studies and the development of molecular models of evolution.

At the Instituto de Investigaciones Biotecnológicas at the Universidad de San Martín, one group has been involved in the sequencing of the Trypanosoma cruzi genome since 1997 [17,18]. This group is also working on the sequencing of different pathogenic bacteria such as Brucella abortus [19] and *Campylobacter fetus,* and common bacteria hosts such as Tupaia belangeri. Currently, with the support of the World Health Organization, this group is involved in the development of potential drug targets for human parasitic diseases using data mining tools with parasite databases [20]. Another genome project of this group is the sequencing of freshwater fish parasite Trypanosoma carassii [21].

In the Instituto Nacional de Tecnología Agropecuaria (INTA), there are several groups working in genomics and microarray data analysis [22]. One of these groups has started a collaboration in the sequencing of the tomato mitochondrial genome as part of the Tomato Sequencing Project [23]. There is also a work in progress toward creating a specific bioinformatics unit to give support to researchers, mainly molecular biology scientists (N. Paniego, personal communication).

Another team involved in genomic analysis is the recently established Centro Regional de Estudios Genómicos (CREG), supported by the Universidad Nacional de La Plata. They have a bioinformatics group, but are focused mostly on information theory and postgenomic analysis. The CREG was part of the sequencing effort of the Rhodnius prolixus genome, but the annotation task was outsourced to the European Biological Institute (EBI). The bioinformatics group at CREG is a truly multidisciplinary team, as it gathers together people from the fields of physics, telecommunications, and astronomy. Also at Universidad Nacional de La Plata, the Instituto de Bioquímica y Biología Molecular (IBBM) runs the local EMBnet node coordinated by Oscar Grau, the first unit dedicated to bioinformatics and computational biology in Argentina, establishing it as a reference center in our country [24].

**Table 1:**
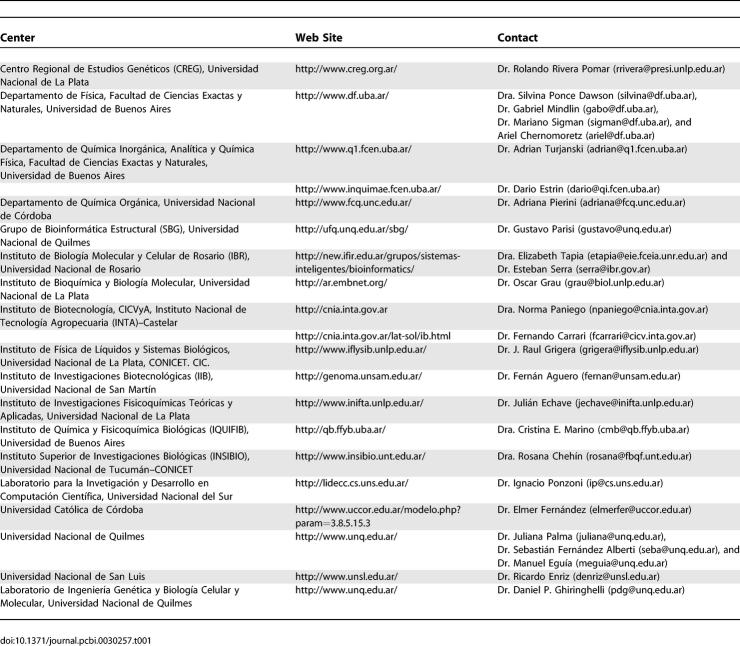
Information about Groups Working on Computational Biology and Bioinformatics in Argentina

A team devoted to microarray data analysis and genome analysis [25], with Elizabeth Tapia and Esteban Serra, is located in the Instituto de Biología Molecular y Celular de Rosario, Universidad Nacional de Rosario. They have developed microarray data error correcting output coding multiclassifiers based on error correcting codes of the recursive type [26].

There are at least four groups working on structural bioinformatics of proteins. Rosana Chehín at the Instituto Superior de Investigaciones Biológicas of the University of Tucumán and CONICET has recently developed a method for clustering large protein families and subfamilies based in a linear array of conserved motifs and domains [27]. Bioinformatics tools such as protein structural alignment and 3-D model quality assessment methods using evolutionary information are being developed in the Structural Bioinformatic Group led by Gustavo Parisi at the Centro de Estudios e Investigaciones, Universidad Nacional de Quilmes. His group uses site-specific substitution matrices [28] derived from simulation using a structurally constrained model of protein evolution [29] to relate protein structure and sequence information contained in a set of aligned homologous proteins. At the same university, a group at the Laboratorio de Ingenieria Genética y Biología Celular y Molecular, led by Daniel Ghiringhelli, works on new strategies for primer design based on information theory.

Adrian Turjanski, at the Departamento de Química Inorgánica, Analítica y Química Física, Facultad de Ciencias Exactas y Naturales, Universidad Nacional de Buenos Aires, focused his research on developing tools using molecular dynamics, quantum mechanics, and molecular mechanics and docking. He has recently been studying the underlying mechanism of phosphorylation and its regulatory role in mitogen-activated protein kinases [30]. Cristina Marino is working in the same field of structural bioinformatics at the Instituto de Química y Fisicoquímica Biológicas (IQUIFIB), Universidad Nacional de Buenos Aires.

The group headed by Elmer Fernández, at the Universidad Católica de Córdoba, mainly focused their research in proteomic and genomic data mining. Also, they perform studies in microarray data analysis, 2-D gel analysis, and statistical biological modeling [31].

## Education and Training

As we mentioned above, scientists who want to work in computational biology should complement their basic degree training taking undergraduate or postgraduate courses. The path to do this is very far from being linear, and the lack of organized information about the minimum necessary knowledge to work in computational biology is very discouraging to new graduates from different areas. Moreover, the lack of information about computational approaches to studying biological systems and also about scientists performing this class of research in Argentina reduces the academic opportunity for new graduates to work in this field. However, there are a few exceptions, such as the degree in bioinformatics launched in 2006 at Universidad Nacional de Entre Ríos (http://www.bioingenieria.edu.ar/).

Although several universities offer biotechnology or biology degrees, very few include in their curricula any computational biology subjects. Universidad Nacional de Quilmes has offered Bioinformatics as an optional course for more than six years, and very recently opened a short specialization on programming. Universidad Nacional de Tucumán offers an Informatics course and others, and Universidad de Buenos Aires offers Introduction to Computational Biology (http://www-2.dc.uba.ar/materias/biocomp/).

The main problem is that most universities are organized into old French-style faculties—i.e., they offer a fixed or somehow limited choice of subjects for each degree. This scheme is used by the most important universities of Argentina. Universities structured into a departmental system integrate their subjects among the different degrees, as is done by Universidad Nacional del Sur and Universidad Nacional de Quilmes.

## Software Development

Most of the aforementioned research teams have developed their own software or adapted source codes from other authors, but in most cases for internal use only. Very few authors have released those programs for public distribution. We think that the main reason for the limited release of software in Argentina is probably related to the deficient computing and programming training of the average Argentinean scientist in the field, as well as to the lack of truly multidisciplinary teams. Also, the universities are, in the best cases, only just starting to discuss licensing policies to release software.

Among the very few examples of public software, we can mention the Linux distribution for bioinformatics called DNALinux [32], which offers a preinstalled set of bioinformatics tools and resources. Also, the local AR.EMBnet node has its own bioinformatics Live CD (http://www.ar.embnet.org/livecd-down.html).

Another system that deserves to be highlighted is wEMBOSS, a Web interface and data manager for the popular EMBOSS software package for biological sequence analysis [33], which was made by the Argentinean EMBnet node in collaboration with the Belgian EMBnet node. The Universidad Nacional de San Martín has released a database for Identification and Ranking of Targets against Neglected Tropical Diseases [34].

Another scientific software program that has been recently released is a program that simulates sequence divergence under structure conservation of protein during evolution (SCPE from structurally constrained protein evolutionary model), developed by Gustavo Parisi and Julián Echave [35]. The program allows the estimation of site-specific substitution matrices that can be used in maximum-likelihood calculations. It has been shown that the SCPE outperforms unconstrained and extensively used models of evolution such as the JTT model [36]. The program also allows the simulation of sequence divergence using proteins with quaternary structure [10]. This program can be obtained from http://ufq.unq.edu.ar/sbg/.

## Computational Biology and Industry

Scientific research is mostly restricted to governmental institutions [37], mainly universities and public institutes. Although the industries and private corporations use computational resources, they mostly import that knowledge from abroad. However, there are some activities related to the use of bioinformatics tools with some applications to agriculture, such as molecular marker–assisted selection for plant and animal breeding.

Agriculture is one of the most prominent industries in Argentina. For this reason, most international biotechnological companies with agricultural interests have one of their branches located in our country. Several of these companies have their own bioinformatics groups, but they are small and their results are kept for internal use.

## Conclusions and Perspectives

The emerging picture of the state of computational biology research in Argentina is characterized by the occurrence of fairly new groups, with limited or no interaction between them. Most of these groups were probably built around the insight of a scientist who had incorporated new disciplines and skills to open new lines of research. Finally, very few of the described groups are truly functioning as multidisciplinary teams. Fortunately, and considering that the critical mass of researchers in the field in Argentina has not yet been reached, Argentinean research projects have explored living systems at multiple organizational levels. We described groups focused on atomic or molecular analysis, sequence, evolution, structure, and dynamics studies (mainly of proteins), and finally groups focused on metabolic and population simulations.

Although interest in the field has increased in the last few years and we expect that the number of researchers and teams will rise outstandingly in the next years, we observe some problems that could hinder the sustainable development of this area in Argentina.

One of the problems we already referred to is the lack of integration of research related to computational biology in Argentina. Possibly, the creation of a specific institution or a society devoted to this activity could consolidate different groups and their interests. This would encourage collaborations and optimize the sharing of resources such as computational capacity or bibliography. It is important to mention that other countries in the region, such as Brazil, Chile, and Mexico, have their own reference centers (for example, the Bioinformatics Laboratory at EMBRAPA from Brazil, http://www.cbi.cnptia.embrapa.br/).

Other problems could be related to economic issues and the lack of financial support from public or private resources to this field. It is important to note that the average investment in research and development in Latin American countries is about 0.2% of the gross domestic product [38]. Although this percentage is clearly below the average research and development spending of developed countries, it is within the range of the countries in the region. When compared within these countries, this investment is shown to be focused in areas other than computational biology. In fact, it is evident that part of the growth in computational biology and bioinformatics results from genome sequencing projects. Brazil has invested not only in automated and sophisticated equipment to obtain sequences, but also in postgenomic informational analysis [39]. A clear example of the varying priority placed on genomics in different countries is shown in the number of automatic sequencers installed in Argentina compared with other countries. By 2003, Argentina possessed five of these sequencers, while at the same time Brazil had more than 50 (C. Parada from Applied Biosystems, personal communication).

There is no specific policy to promote computational biology in Argentina, but there is a plan to promote biotechnology, including bioinformatics (L. Barañao, Head of Agencia Nacional de Promoción Científica y Tecnológica, personal communication) [40]. We think that policies using public resources should promote the creation of institutions to integrate technological, financial, and academic resources, as is being done at INTA.

Not all the problems are economic. The demand for computer scientists to work in close relation with experimental groups has progressively increased in the last two years. The problem that arises here, related to academic issues, is that universities, with the exception of Universidad Nacional de Entre Ríos, do not realize the value of computational biology either for its intrinsic academic significance or as a source of jobs for new graduates. To remedy this, biologically oriented degrees should include more flexible curricula to allow for the existence of different degree orientations.

Summing up, we think that there is a lot of work ahead to achieve sustainable growth of computational biology in Argentina. The quality of the academic production of the groups described, the existence of innovative basic research lines, and the study of systems with regional interest indicate a promising future. Together they reveal that in Argentina the field of computational biology is only just beginning. 

## Abbreviations

CONICET: Consejo Nacional de Investigaciones Científicas y Técnicas
CREG: Centro Regional de Estudios Genómicos
INTA: Instituto Nacional de Tecnología Agropecuaria
SCPE: structurally constrained protein evolutionary model
